# Total saponins from Lilium lancifolium: a promising alternative to inhibit the growth of gastric carcinoma cells

**DOI:** 10.7150/jca.42285

**Published:** 2020-04-27

**Authors:** Yin-Yu Zhang, Lin-Ming Luo, Yu-Xiang Wang, Neng Zhu, Tan-Jun Zhao, Li Qin

**Affiliations:** 1 Division of Stem Cell Regulation and Application, School of Pharmacy, Hunan University of Chinese Medicine, Changsha, China; 2 The First Hospital of Hunan University of Chinese Medicine, Changsha, China

**Keywords:** Total saponins from Lilium lancifolium, proliferation, apoptosis, migration, Gastric Carcinoma Cells

## Abstract

*Bulbus Lilii*, as a medicinal and edible plant, has anti-inflammatory, anti-oxidative and immunopotentiating pharmacological activities, which seems to be therapeutic on cancer prevention. The purpose of this study was to investigate the effects of total saponins from Lilium lancifolium (TSLL) on proliferation, apoptosis and migration of human gastric carcinoma cells lines SGC-7901 and HGC-27 and its underlying mechanism. The results showed that TSLL inhibited the proliferation of gastric carcinoma cells by suppressing the level of proliferating cell nuclear antigen (PCNA) and increased p21 level. TSLL induced cells apoptosis by up-regulating expression of pro-apoptotic protein Bax and down-regulating anti-apoptotic protein Bcl-2 expression. Meanwhile, TSLL remarkably inhibited cell migration and invasion, decreased matrix metalloproteinase-2 (MMP-2) expression and increased tissue inhibitor of metalloproteinases-1 (TIMP-1) expression. Notably, TSLL had stronger anti-cancer effect on undifferentiated HGC-27 cells than differentiated SGC-7901 cells. Accordingly, TSLL might be a promising candidate to prevent and suppress the growth of gastric carcinoma cells.

## Introduction

Gastric carcinoma is one of the most common malignant cancers, and its mortality ranks third in the world in cancer 1. The majority of patients with gastric carcinoma are diagnosed in the advanced stage due to the lack of early diagnosis. Clinically, the most frequent cause of treatment failure following surgery for gastric carcinoma is peritoneal dissemination, mainly caused by the seeding of free cancer cells from the primary gastric carcinoma, which is the most common type of spread 2. Moreover, its high recurrence rate and high metastasis rate are the most important reasons for the low survival rate of gastric carcinoma patients 3. Therefore, effective drugs capable of preventing or treating gastric carcinoma are needed urgently to improve the therapeutic outcomes. Herein, we attempted to develop a new compound from natural products against gastric carcinoma to avoid the side effects, such as difficult to diagnose and high metastatic potential.

*Bulbus Lilii*, from a kind of perennial bulbiferous plant in the genus Lilium of the family Liliaceae, not only has high nutritional value with rich protein, dietary fiber and vitamins, is also rich in selenium, calcium, magnesium, zinc and other trace elements. As a traditional Chinese medicine, *Lilii* mainly contains steroidal saponins, alkaloids, organic acids and polysaccharides 4. Steroidal saponins have been identified in lily bulbs 5. As one of the main biological active ingredients in lily, it qualified with many biological activities, including anti-inflammatory, anti-bacterial, anti-oxidation 6. However, up to date, there are few reports on the anticancer activity of Total saponins of *Lilium lancifolium* (TSLL). Our previous study found that TSLL inhibited the proliferation, migration and invasion of lung cancer A549 cells lines and induced apoptosis. Here, we explored the effects and potential mechanism of TSLL on gastric carcinoma cells, so as to provide a new strategy for the treatment of gastric carcinoma patients.

## Materials and methods

### Chemicals

Dulbecco's Modified Eagle Medium (DMEM), fatal bovine serum (FBS), 0.25% trypsin-EDTA, and phosphate buffered saline (PBS) were purchased from Gibco (Grand Island, NY, USA). Matrigel was purchased from BD Biosciences (San Jose, CA, USA). Bovine serum protein (BSA), fibronectin (FN), and 0.1% Crystal Violet staining solution were purchased from Solarbio (Beijing, China). The cell counting kit-8 (CCK-8) was purchased from Dojindo Laboratories (Kumamoto, Japan). YF488 Click-iT EdU imaging kit, YF488-Annexin V/PI apoptosis detection kit, and Hoechst 33342 were obtained from US Everbright (Silicon Valley, USA). Anti-PCNA, anti-P21, anti-Bcl-2, anti-Bax, anti-MMP-2, anti-TIMP-1, anti-E-cadherin, and anti-GAPDH rabbit polyclonal antibodies were purchased from Proteintech Group (Chicago, IL, USA). Anti-activated-Casepase-3 and anti-Vimentin rabbit polyclonal antibodies were purchased from Bioworld Technology (St. Louis Park, MN, USA).

### Sample Preparation

**Figure 1:**
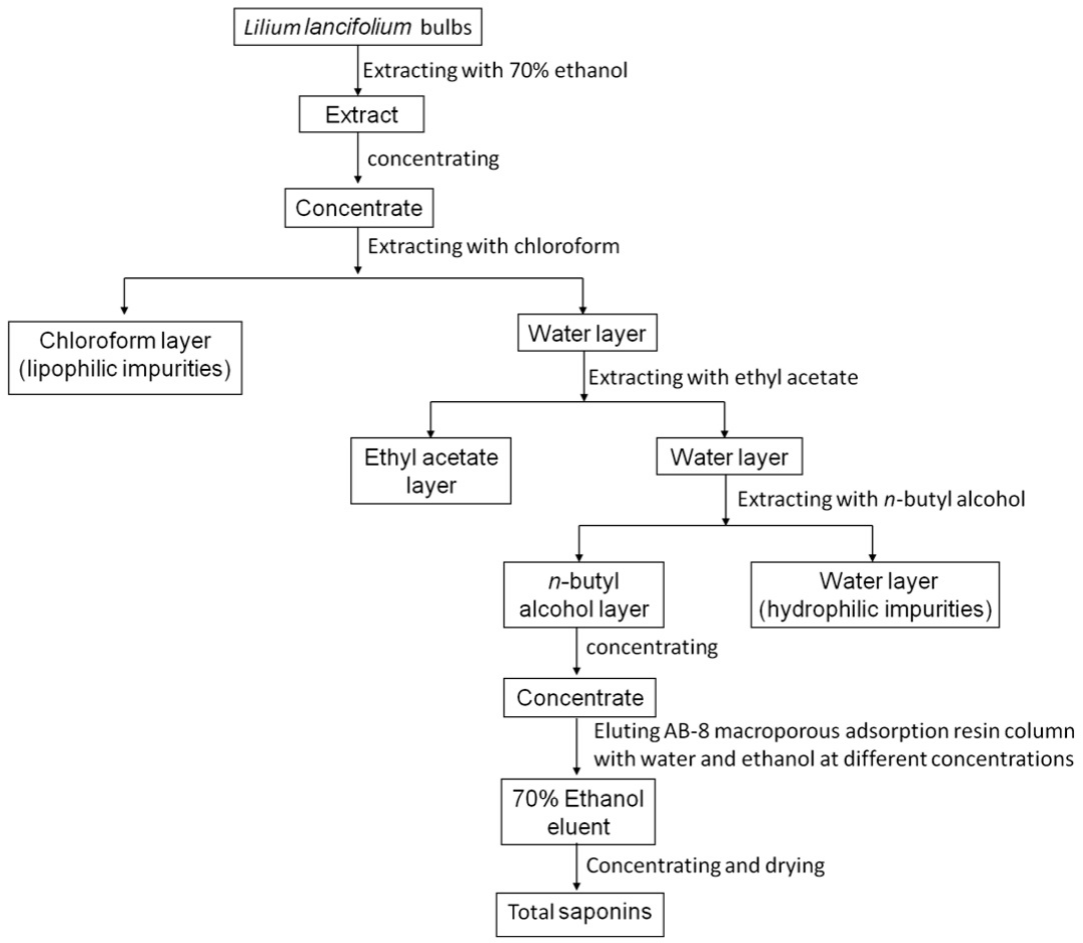
The flow of TSLL sample preparation.

The fresh bulbs of *Lilium lancifolium* Thunb (provided by Yao Sheng Tang (Hunan) Pharmaceutical Co., Ltd.) were extracted with 70% ethanol and concentrated by heating. To obtain the concentrated extracts, the solution was repeatedly extracted with chloroform, ethyl acetate and *n*-butanol in turn. Each round lasted for 2h until the water layer is almost colorless. Then, the solution was concentrated by a rotary evaporator and dried by evaporating dish. The crude extract was further purified by using column chromatography. The dried *n*-butanol extract was dissolved in distilled water and filled into a prepared low-pole AB-8 macro-porous adsorption resin column. The column was successively eluted with 4 L distilled water, 10%, 30%, 50%, 70% and 100% ethanol. The 70% ethanol eluent was collected, concentrated and dried to obtain solid total saponins extract (Fig. 1). TSLL was a test sample and cisplatin (CDDP) was used as a reference to compare the anti-cancer effect of TSLL.

### Cell culture and treatment

Human gastric carcinoma cell lines SGC-7901 (moderate differentiated) and HGC-27 (undifferentiated) were purchased from the Cell Bank of the Chinese Academy of Sciences (Shanghai, China), and were cultured at 37^o^C and humidified 5% CO_2_ incubator with DMEM high glucose medium containing 10% FBS, 100 mg/mL penicillin, and 100 mg/mL streptomycin. Culture medium was changed every 2-3 days.

### Cell viability assay

SGC-7901 or HGC-27 cells in the logarithmic growth period were inoculated in 96-well culture plate at density of 1×10^5^ cells/mL. After incubation in a humidified incubator with 5% CO_2_ at 37^o^C for 24 h and added starvation for 12 h, different concentration of TSLL (0, 25, 50, 100, 200 and 400 μg/mL) and CDDP (4 μg/mL) were used to treat cells for 24 h, 48 h and 72 h, respectively. In addition, cells with complete medium containing 0.3% ethanol were served as negative control. After treatment, the medium was removed, 100 μL 10% CCK-8 solution was added, and cells were incubated with CCK8 for 50 min. The optical density (OD) of yellow formazan product was measured by using a microplate reader (Elx 800, BioTek, USA) at 450 nm and generated by control taken as 100%.


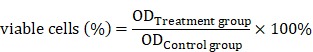


### Clone formation assay

SGC-7901 and HGC-27 cells (300 cells/well) were planted into 6-well culture plate with DMEM medium for 24 h and were treated with various concentrations of TSLL (0, 25, 50, 100, 200, 400 µg/mL) or 0.3% ethanol as a vehicle control. The growth condition of cells was observed every 2 days under microscope, and the medium was renewed every 4 days until the visible clones appeared. The medium was discarded and the cells were carefully washed with PBS twice. After being fixed with methanol for 15 min, the cells were stained with 0.1% crystal violet dyeing solution for 15 min before washing with PBS and air-drying. The clones with more than 50 cells were counted with an ordinary optical microscope and the clone formation rate was calculated with the following formula:


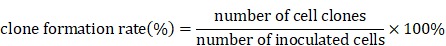


### EdU staining

A Click-iT EdU kit was used according to the manufacturer's instructions. SGC-7901 and HGC-27 cells at a density of 1×10^4^ cells/well were seeded in 96-well plates with TSLL (25, 50, 100, 200 and 400 µg/mL), while the control well substituted 0.3% ethanol for drug. Then, 50 μmoL EdU solutions were added to the cells, and incubated at 37°C for another 2 h. After collection, the cells were fixed with 4% paraformaldehyde for 15 min, and infiltrated by using Triton X-100 solution for 20 min at room temperature in the dark. In order to detect cells proliferation, the fixed cells were then stained with Click-iT reaction mixture and cell nuclei were stained with 1 μg/mL DAPI in the dark at room temperature for 30 min. The stained cells were observed under an inverted fluorescence microscope, and the fluorescent cells were counted by using Image Pro Plus 6.0 (IPP). The DNA synthesis rate was calculated by using following equation:


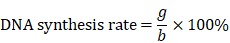


(g represents the number of green fluorescent cells, and b represents the number of blue fluorescent cells)

### Annexin V/PI double staining

Annexin V/propidium iodide (PI) double staining assay was done based on the manufacturer's instructions. In brief, SGC-7901 and HGC-27 cells were planted into 6-well culture plate at a density of 1.0×10^5^ cells per well, and pre-incubated in an incubator of 37^o^C, 5% CO_2_ for 24 h. Different concentrations of TSLL (100, 200, 400 µg/mL) and CDDP (10 µg/mL) were added to corresponding well while removing the original medium. Additionally, control well was treated with 0.3% ethanol. After 36 h of treatment, medium was removed and cells were immobilized with 4% polyoxymethylene for 15 min. Annexin V/PI double staining was used to determine apoptosis.

### Hoechst 33342 staining

In order to investigate the cells apoptosis, morphological analysis was performed by Hoechest 33258 staining. SGC-7901 and HGC-27 cells were seeded in a 96-well tissue culture plate at a density of 1×10^4^ cells/well. Cells were treated with different concentrations of TSLL (100, 200, 400 µg/mL and CDDP (10 µg/mL), and control well was treated with 0.3% ethanol. After 36 h of treatment, the cells were incubated with 100 μL Hoechst 33258 at 37^o^C for 20 min, followed by observation under a fluorescence microscope. Strong fluorescence can be observed in the nuclei of apoptotic cells, while weak fluorescence was observed in non-apoptotic cells.

### Wound healing assay

SGC-7901 and HGC-27 cells were seeded into 6-well culture plate for 24 h and the cells were followed by starvation overnight. Wounded by scratching with white pipette tips, the cells were incubated with condition medium containing 1% FBS and then received various treatments of TSLL (0, 25, 50, 100 and 200 µg/mL). The number of migrated cells was determined under an inverted microscopy at 6 h, 12 h and 24 h.

### Transwell migration assay

*In vitro* invasion assay was performed by using transwell plates (Guangzhou Jet Bio-Filtration, Co., Ltd) with 8 µm pores. The cells (1×10^6^ cells) with TSLL (0, 25, 50, 100 and 200 µg/mL) and serum-free DMEM medium were added to the upper chamber of the transwell plates that were pre-coated with matrigel. Then DMEM medium containing 10% FBS as a chemo-attractant was added to the lower chamber. After incubation for 48 h, cells on the upper surface were removed by using cotton wool and the rest cells were fixed with methanol and stained with 0.5% crystal violet. Images were captured and the cells were counted by measuring the optical density (OD) value of each well at 570 nm with a microplate reader.

### Cell adhesion assay

The 96-well plates were coated with 50 μL fibronectin at 4°C for 6 h, and then washed twice with PBS and blocked with serum-free DMEM+2% BSA for 30 min at 37^o^C. SGC-7901 and HGC-27 cells were treated with different concentrations of TSLL (0, 25, 50, 100 and 200 μg/mL) for 24 h at 37^o^C in a humidified incubator supplemented with 5% CO_2_. To remove the non-adherent cells, plates were gently washed twice with PBS. Then, 100 μL of DMEM medium and 10 μL CCK-8 solution were added to each well. After incubation for 50 min, the OD at 450 nm of each well was measured with a microplate reader. The cell adhesion ratio was calculated according to the following formula:


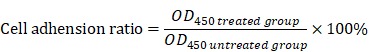


### Western blotting

Western blotting was used to detect the proteins that were related to cell proliferation, apoptosis and invasion and metastasis in gastric carcinoma cells SGC-7901 and HGC-27. Cells were harvested and lysed in cell-lysis buffer. Protein concentrations were quantified by a BCA protein assay according to the manufacturer's instructions. Twenty micrograms of each sample were separated by 12% (v/v) SDS-PAGE gel, and then the protein samples were transferred onto polyvinylidene difluoride (PVDF) membranes. The resultant membrane was incubated with Tris-buffered saline and Tween-20 (TBST) containing 5% skim milk for blocking for 2 h. Membranes were incubated at 4°C overnight with primary antibody (1:1000) and washed three times with TBST buffer. The membranes were then incubated with the horseradish peroxidase-conjugated secondary antibody at room temperature for 2 h. Protein bands were visualized using ECL reagent.

### Statistical analysis

All values were presented as the mean ± SD, and statistical analyses were performed using GraphPad Prism 7 (GraphPad, San Diego, CA, USA). A one-way analysis of variance (ANOVA) was employed to analyze the differences within multiple groups and *P* < 0.05 was considered significant.

## Results

### TSLL has cytotoxic effects on human gastric carcinoma cells

**Figure 2:**
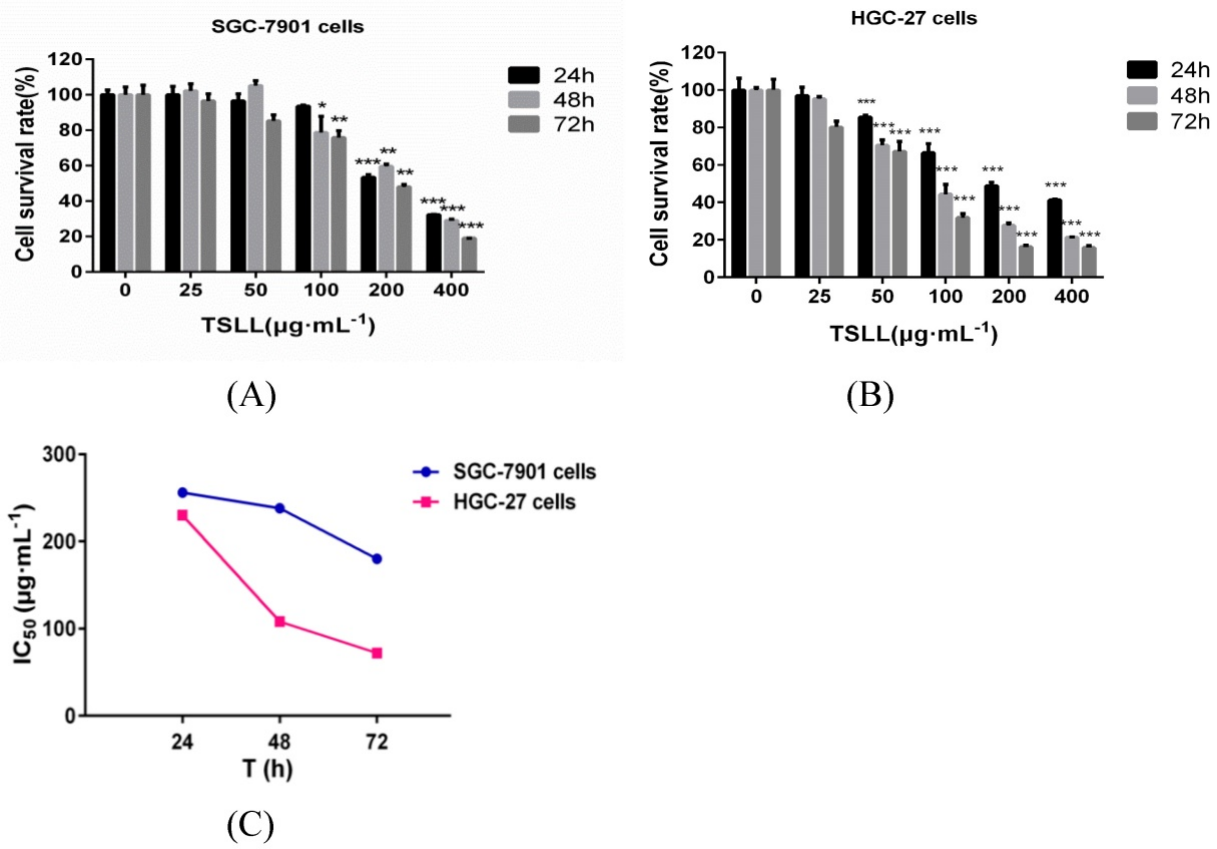
The effect of TSLL on gastric carcinoma cells viability. To determine whether TSLL exhibited cytotoxic effects on gastric carcinoma cells, SGC-7901 (A) and HGC-27 cells (B) were treated with different concentration (0, 25, 50, 100, 200 and 400 μg/mL) and CDDP (4 μg/mL), CCK8 assay was performed at 24, 48 or 72 h after treatment. The IC50 values were calculated (C). The values represent the means
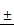
SD of at least three independent experiments, ^*^p < 0.05, ^**^p < 0.01, ^***^p < 0.001.

The total saponins from the fresh bulbs of Lilium lancifolium Thunb were isolated successfully. To explore the role of TSLL in different stages of gastric carcinoma, SGC-7901 and HGC-27 cells were applied in this study 7. We found that TSLL could significantly inhibit the survival rate of SGC-7901 cells at the concentration of 200 and 400 μg/mL (*P* < 0.001) (Fig. 2A), and had the similar inhibitory effect on HGC-27 cells (*P* < 0.001) at 50-400 μg/mL TSLL (Fig. 2B). Meanwhile, 100-400 μg/mL TSLL treatment for 48 h or 72 h could significantly inhibit SGC-7901 cells survival rate (*P* < 0.05, *P* < 0.01, *P* < 0.001), while 50-400 μg/mL TSLL had the similar effect on HGC-27 cells. Thus, TSLL inhibits the proliferation of SGC-7901 and HGC-27 cells in a time- and dose-dependent manner. Notably, the inhibitory effect of TSLL on HGC-27 cells was stronger than that of SGC-7901 cells (Fig. 2C). These data indicate that HGC-27 cells appeared to be more sensitive to TSLL than SGC-7901 cells.

### TSLL inhibits the proliferation of gastric carcinoma cells

**Figure 3:**
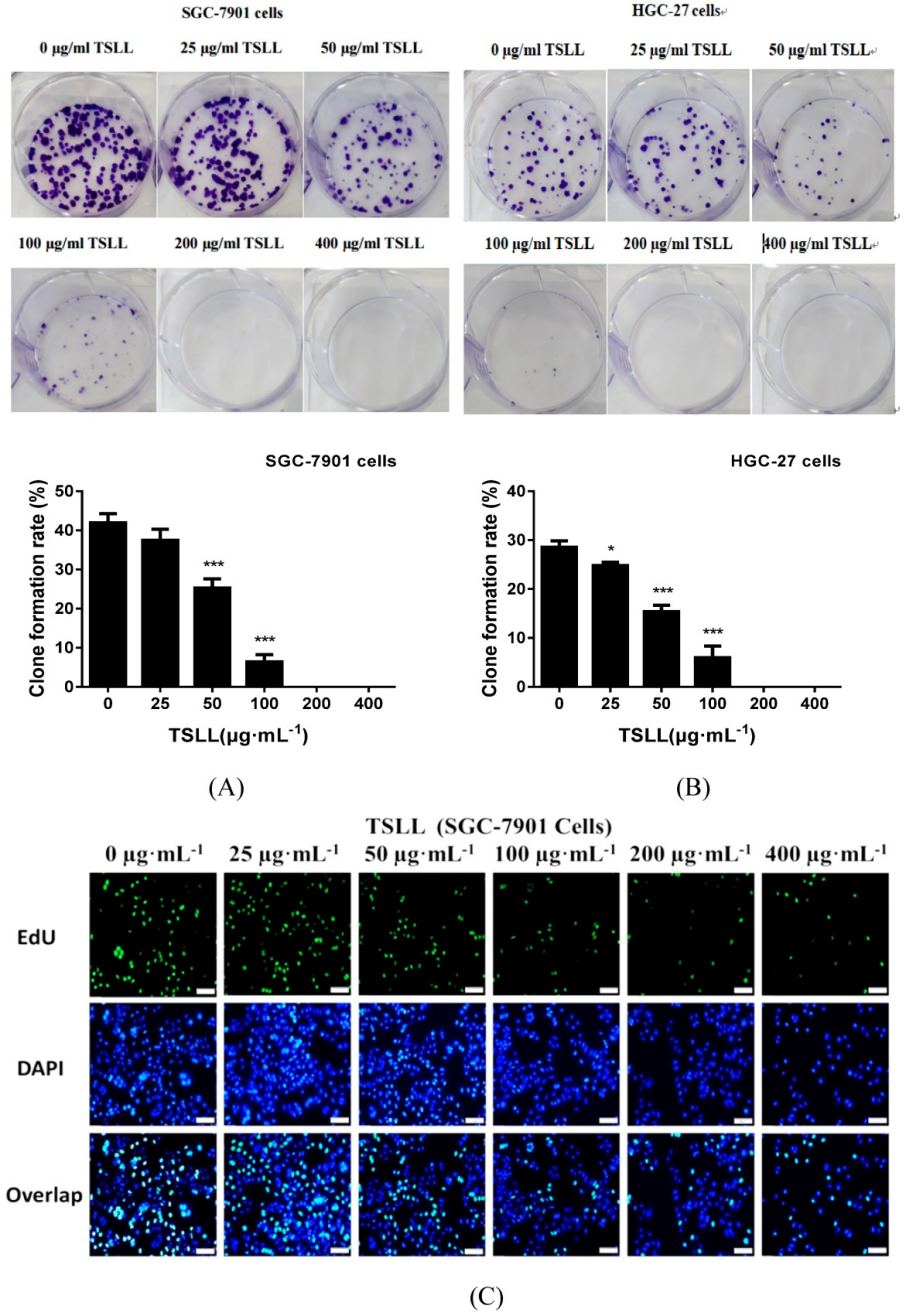

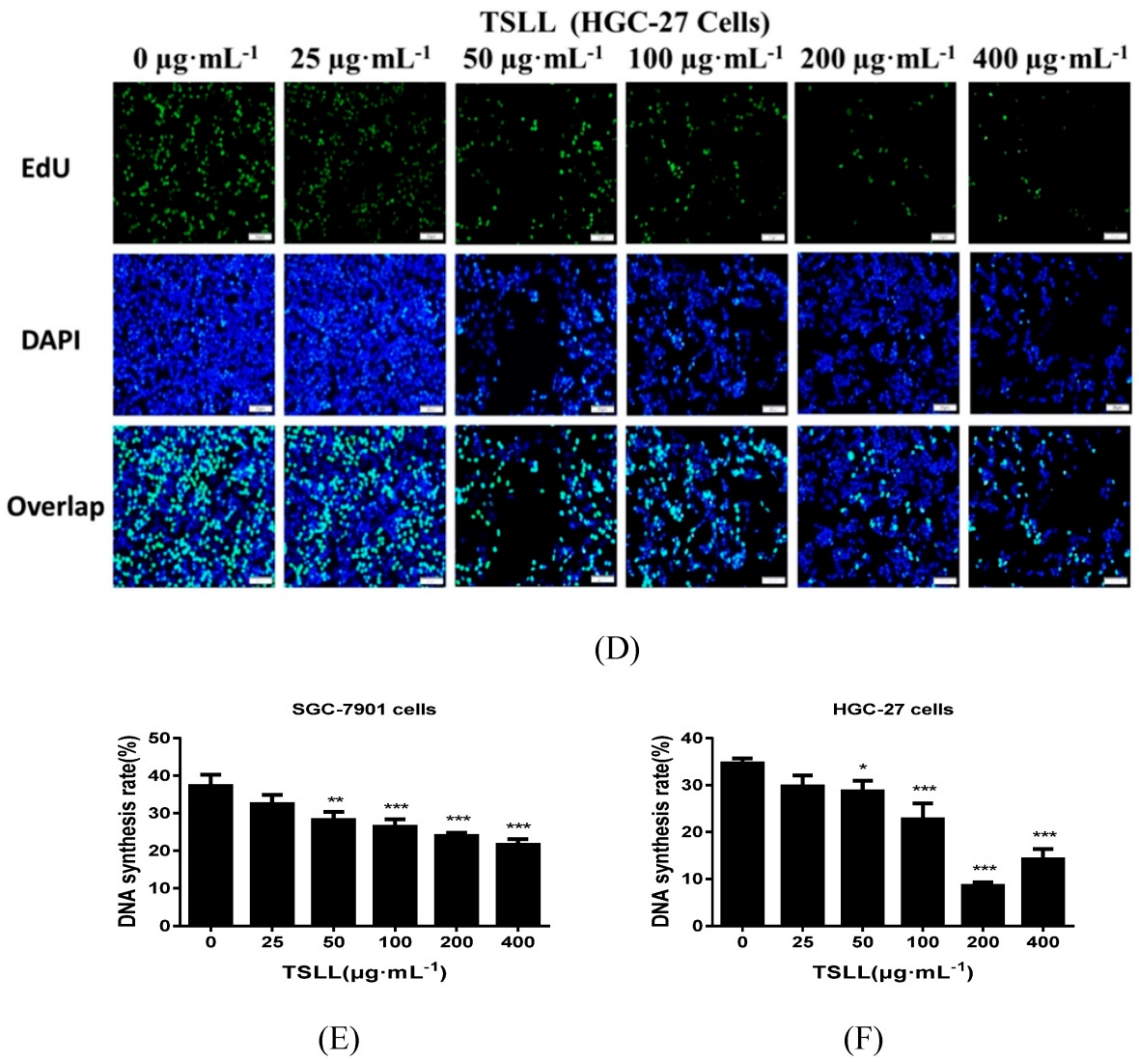
TSLL inhibited the proliferation and colony formation of gastric carcinoma cells. TSLL treatment reduced colony formation of SGC-7901 (A) and HGC-27 cells (B). After TSLL treatment for 24 h, viable cells were counted and the relative clone formation rate was calculated according to the ratio of the number of cell clones to total number of inoculated cells. EdU staining was used to determine DNA synthesis in SGC-7901 (C) and HGC-27 cells (D). The fluorescence was observed under inverted fluorescence microscope after Edu staining. Quantification of DNA synthesis was analyzed (E, F). The data represented means
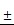
SD. ^*^p < 0.05, ^**^p < 0.01, ^***^p < 0.001, and the difference between TSLL-treated groups and the control was statistically significant.

As shown in Fig. 3, TSLL significantly reduced the proliferation ability of gastric carcinoma cells. Compared to the control group, TSLL also dramatically inhibited the clone formation of SGC-7901 cells at the concentration range of 50-400 μg/mL (*P* < 0.001) (Fig. 3A). There was almost no colony formation in the 200-400 μg/mL TSLL-treated group, suggesting that TSLL not only inhibited the proliferation of SGC-7901 cells, but also induced cell death or exerted direct cytotoxicity at high concentrations. TSLL also showed the similar effect on HGC-27 cells (Fig. 3B). Furthermore, the DNA synthesis rate of SGC-7901 (Fig. 3C) and HGC-27 (Fig. 3D) cells was inhibited by TSLL in a dose-dependent manner. Undoubtedly, the effect of TSLL on DNA synthesis of SGC-7901 and HGC-27 cells was different. 400 μg/mL TSLL exhibited the maximum DNA synthesis inhibition rate of 15.60% in SGC-7901 cells, while 26.15% in HGC-27 cells. In line with previous results, the inhibitory effect of TSLL on HGC-27 cell proliferation is stronger than that on SGC-7901 cells (Fig. 3E and 3F).

### TSLL inhibits the proliferation of gastric carcinoma cells by down-regulating PCNA expression and promoting p21 expression

**Figure 4:**
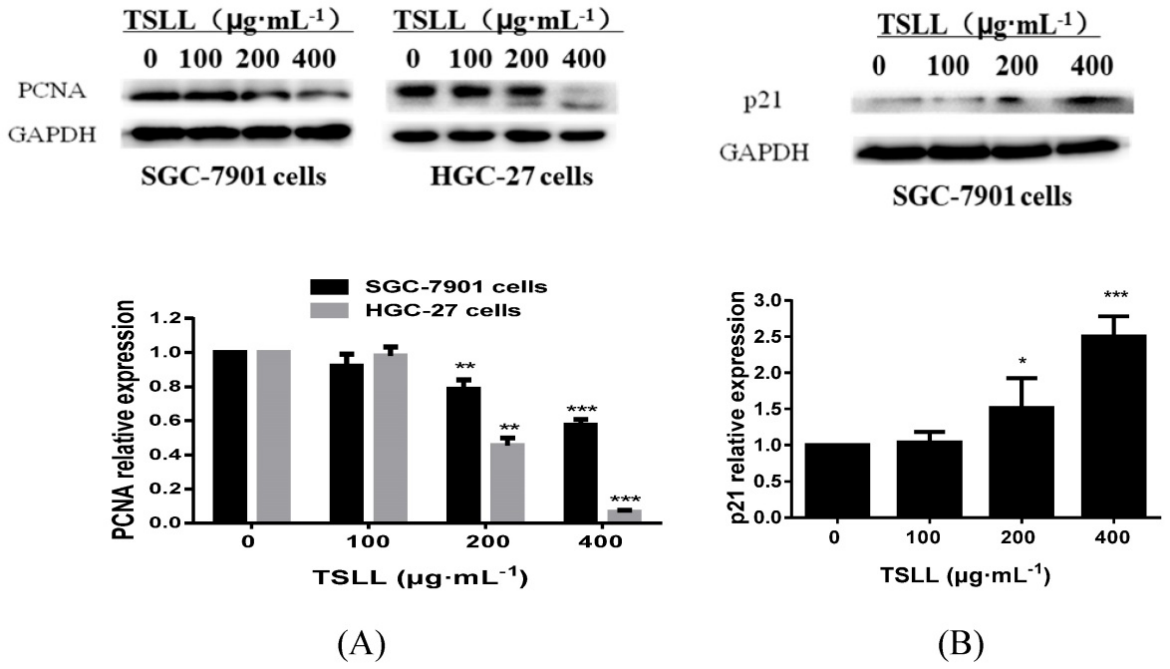
TSLL up-regulated p21 and down-regulated PCNA expression in gastric carcinoma cells. The expression of PCNA was detected by Western blotting analysis (A). The expression of p21 was also determined in SGC-7901 cells treated with TSLL (B). Quantitative results of PCNA and p21 levels, which were adjusted with GAPCH protein level. The data represent means
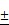
SD. ^*^p < 0.05, ^**^p < 0.01, ^***^p < 0.001, significant difference between TSLL-treated groups and the control.

It is known that proliferating cell nuclear antigen (PCNA)/p21 signaling pathway is one of the key pathways for cell replication. As a cyclin-dependent kinases (CDKs) inhibitor protein, p21 can not only regulate cell-cycle, but also participate in cells apoptosis. These effects are largely dependent on the antagonistic effects between p21 and PCNA. For example, p21 inhibits the synthesis of DNA by directly interacting with PCNA binding proteins and competing for PCNA, which is required in DNA repairing 8, 9. Herein, we found that TSLL (200 and 400 μg/mL) inhibited the expression of PCNA in both SGC-7901 cell lines and HGC-27 cell lines. Additionally, as shown in Fig. 4B, TSLL (200 and 400 μg/mL) treatment significantly increased the expression of p21 in SGC-7901 cells (*P* < 0.05, *P* < 0.001).

### TSLL induced apoptosis of gastric carcinoma cells

**Figure 5:**
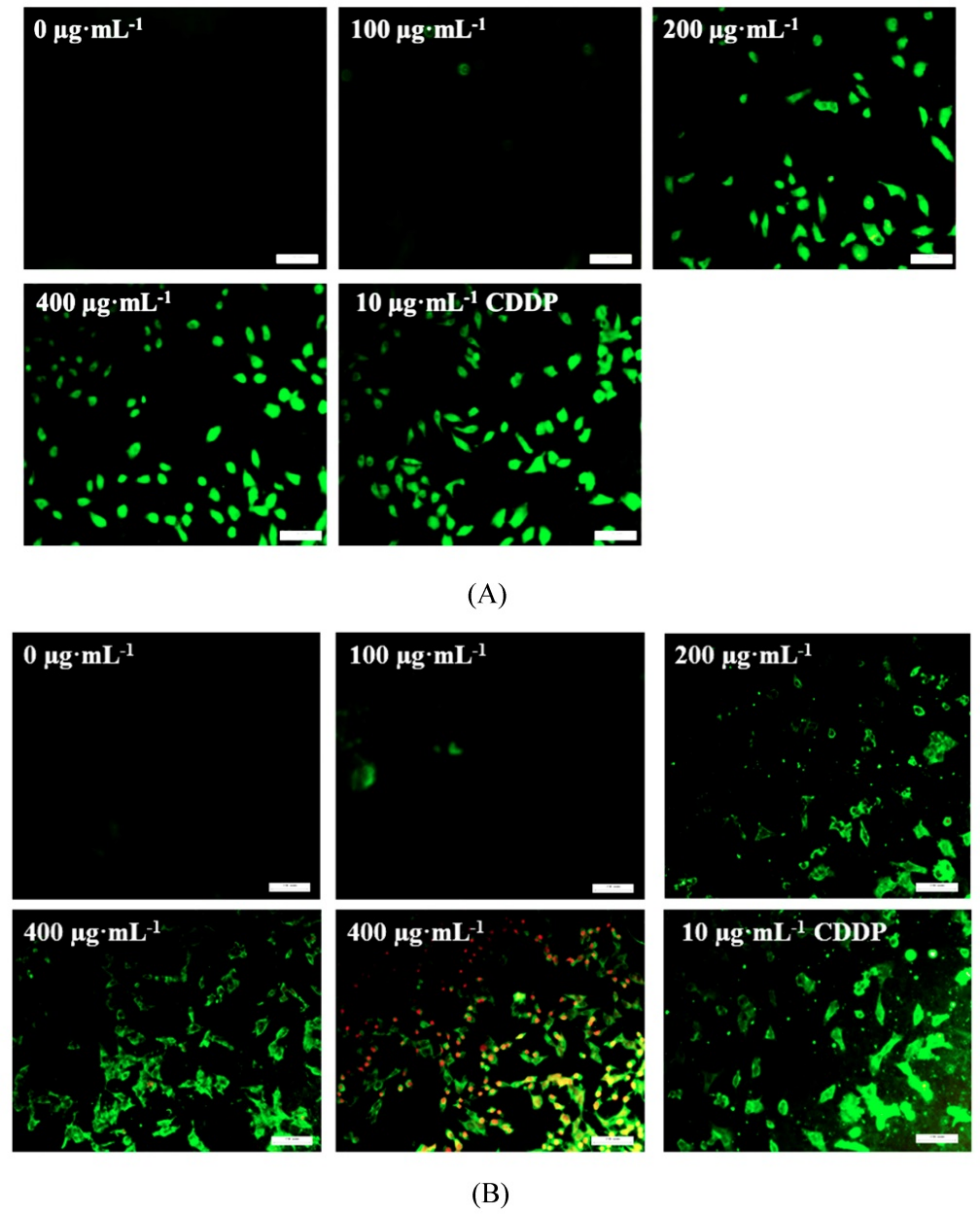

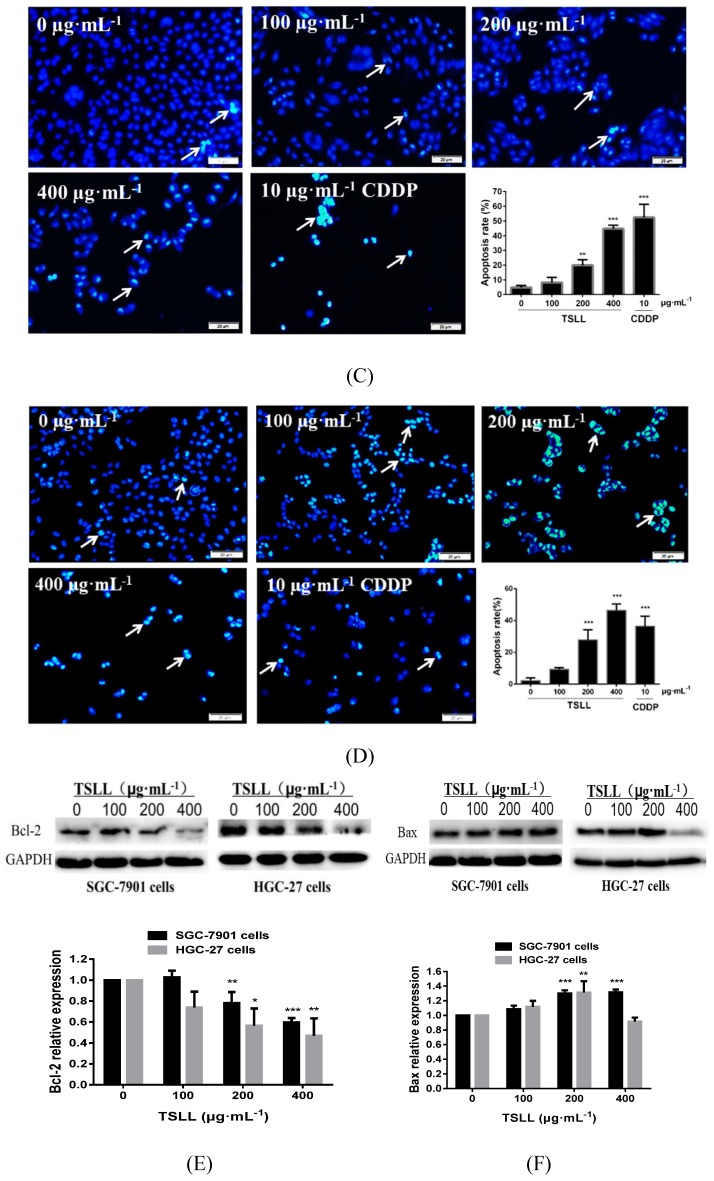
TSLL induced cells apoptosis in gastric carcinoma cells. Annex V/PI double staining assay was applied to detect the apoptotic cells in SGC-7901 (A) and HGC-27 (B) cells. The effect of TSLL on apoptosis in SGC-7901 (C) and HGC-27 (D) cells was examined by using Hoechst 33342 staining. Fragmented nuclei were emphasized by white arrows. Western blotting was performed to analyze the protein levels of Bcl-2 (E) and Bax (F) in SGC-7901 and HGC-27 cells. GAPDH was used as a loading control. The data represent means
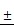
SD. ^*^p < 0.05, ^**^p < 0.01, ^***^p < 0.001, significant difference between TSLL-treated groups and the control.

As shown in Fig.5, TSLL (200 and 400 μg/mL) treatment in SGC-7901 and HGC-27 cells for 36 hours induced numerous amount of apoptotic cells with strong green fluorescence (Fig. 5A and 5B). It is worth noting that the green fluorescence density in 400 μg/mL TSLL treatment group was significantly higher than that in 200 μg/mL TSLL treatment group. These results indicate that high concentrations of TSLL can activate the apoptotic pathway in gastric carcinoma cells. Meanwhile, Hoechst 33342 staining showed TSLL treatment induced typical apoptotic phenotypic changes, such as chromatin condensation, nuclear condensation and even cleavage, especially in SGC-7901 cells treatment. The apoptotic cell rates of the three groups with different concentration of TSLL (100, 200 and 400 μg/mL) were 8.11%, 19.90% (*P* < 0.01) and 44.75% (*P* < 0.001), respectively. Similar results were also observed in HGC cells treated with TSLL (100, 200 and 400 μg/mL), and the apoptotic cell rates of three group were 9.37%, 27.90% (*P* < 0.001) and 46.46% (*P* < 0.001), respectively.

**Figure 6:**
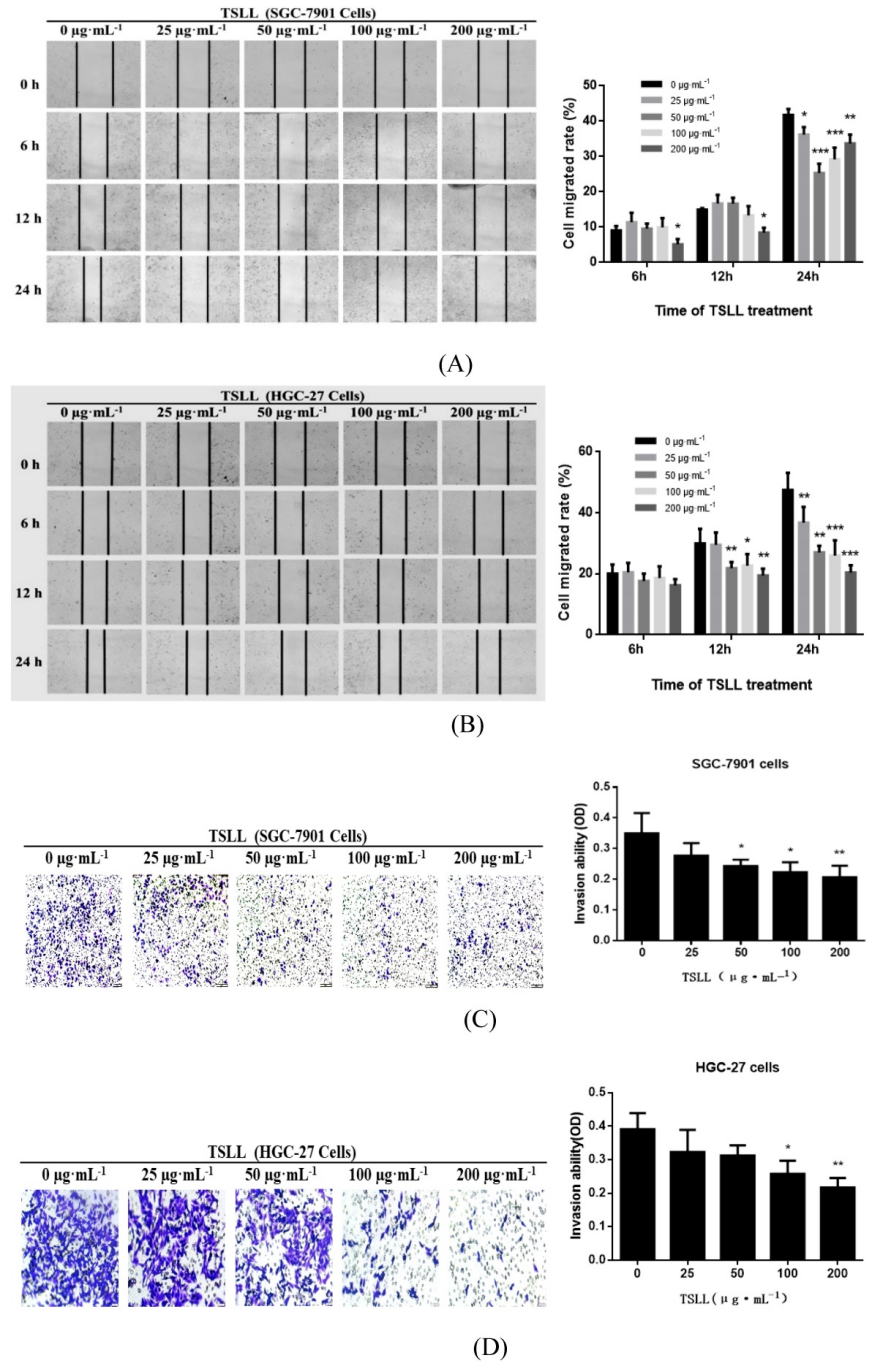
TSLL inhibited migration and invasion in the gastric carcinoma cells. Wound healing assays in SGC-7901 (A) and HGC-27 cells (B) after TSLL (0-200 μg/mL) treatment for 24 h. The inhibitory effect of TSLL on SGC-7901 (C) and HGC-27 cells (D) invasion was detected by a transwell assay. Representative images showed invasion of matrigel membranes stained with 0.5% crystal violet. The data represent means
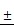
SD. ^*^p < 0.05, ^**^p < 0.01, ^***^p < 0.001, significant difference between TSLL-treated groups and the control.

As we know, Bcl-2 family proteins (Bcl and Bax) serve as important regulators involved in apoptosis 10. And the equilibrium between pro-apoptotic and anti-apoptotic Bcl-2 family proteins is an essential element of the cell apoptosis. As shown in Fig. 6, the levels of Bcl-2 in SGC-7901 and HGC-27 cells decreased after TSLL (200 and 400 μg/mL) treatment, compared to the control cells. In addition, the level of Bax was up-regulated both in SGC-7901 and HGC-27 cells with TSLL treatment (Fig. 5C and 5D).

### TSLL inhibits migration and invasion of gastric carcinoma cells

Migration and invasion are key steps during the development of cancer. In the present study, wound healing assay showed that the inhibitory effects of TSLL on migration increased in a dose-dependent manner in SGC-7901 and HGC-27 cells after TSLL treatment for 12 and 24 h (Fig. 6A and 6B). Consistent with the migration results, TSLL also impaired the capability of SGC-7901 and HGC-27 cells to pass through the artificial basement membrane significantly (*P* < 0.05, *P* < 0.01).

### TSLL promotes TIMP-1 expression and suppresses MMP-2 expression in gastric carcinoma cells

**Figure 7:**
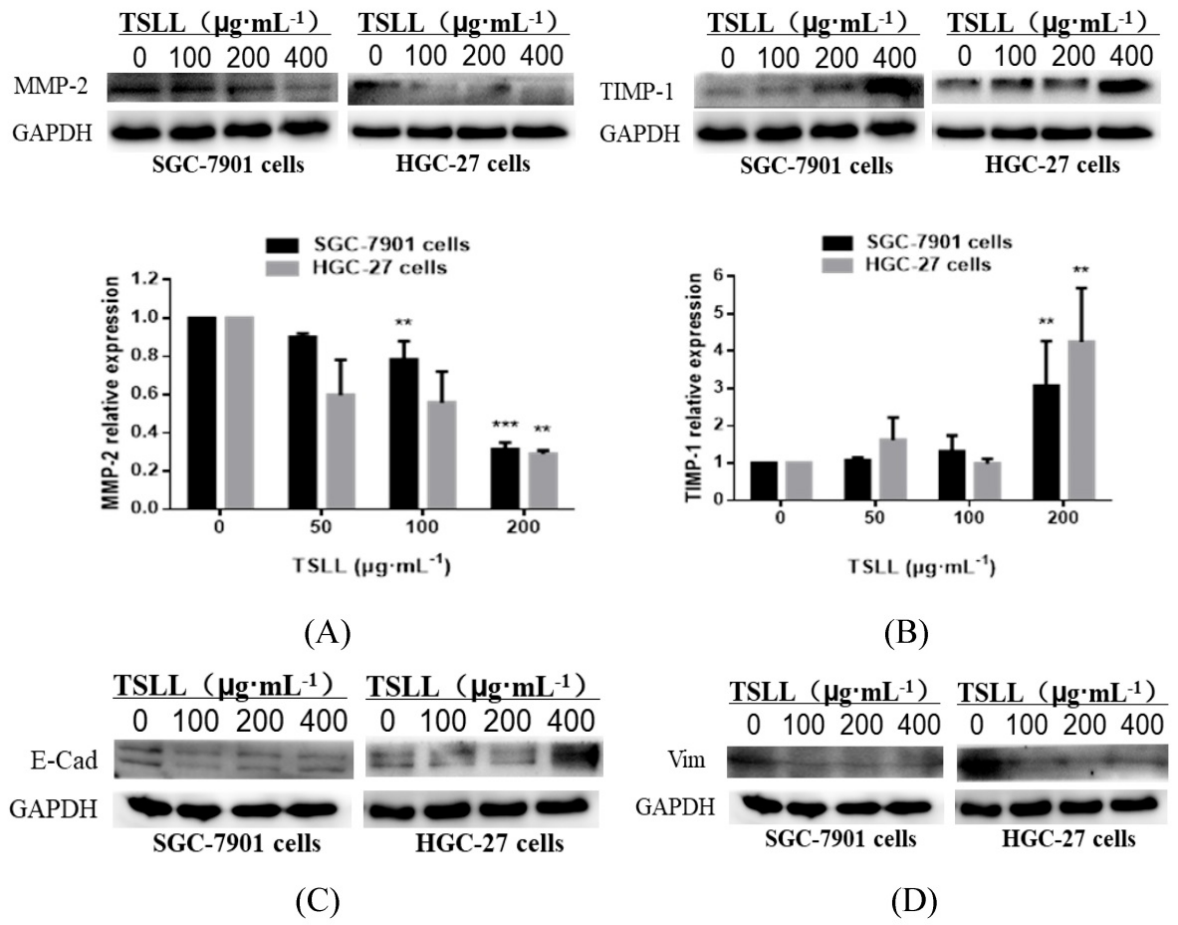
Analysis of the protein levels of MMP-2 (A), TIMP-1 (B), E-Cad (C) and Vim (D) in SGC-7901 and HGC-27 cells by Western blotting. Quantitative results of protein levels, which were adjusted with GAPDH protein level. The values represented the protein expression levels of at least three independent experiments. The data represent means
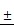
SD. ^**^p < 0.01, ^***^p < 0.001, significant difference between TSLL-treated groups and the control.

The invasion and metastasis of gastric carcinoma cells were closely related to the abnormal expression of matrix metalloproteinases (MMPs) 11, 12 and its inhibitory factors (TIMPs) 13. The data showed that TSLL treatment led to a reduction of MMP-2 expression (Fig. 7A) and an increase of TIMP-1 expression (Fig. 7B) in SGC-7901 and HGC-27 cells. Generally, malignant tumors, especially epithelial tumors, often undergo epithelial-mesenchymal transformation (EMT) to promote the invasion and metastasis of cancers 14, 15. EMT confers vital properties to cancer cells for invasion and metastatic dissemination, especially increased motility, invasiveness and the ability to degrade components of the extracellular matrix (ECM) 16. We further examined the effect of TSLL treatment on the expression of EMT-related proteins in gastric carcinoma cells. Unexpectedly, TSLL intervention had no significant effect on the expression of E-Cad (Fig. 7C) and Vim (Fig. 7D) protein in SGC-7901 and HGC-27 cells. The results implied that TSLL might inhibit the cancer invasion and metastasis through mechanisms other than EMT, such as reducing the accumulation of cancer stem cells, maintaining cancer microenvironment homeostasis and so on. The detailed mechanism needs further investigating.

## Discussion

Cancer remains one of the deadliest diseases in the world and chemotherapy is still a major strategy. However, the efficacy of chemotherapy is often limited by adverse effects in normal tissues, especially serious toxicities and drug resistance 17, 18. An increasing number of anti-cancer compounds from natural products provide a promising alternative to the chemoprevention and chemotherapy because of their favored safety and multi-target effects 19. In the present study, we applied the extraction and purification of TSLL and firstly demonstrated that TSLL could effectively suppress gastric carcinoma cells proliferation and metastasis, and induce cells apoptosis. TSLL inhibited the proliferation of gastric carcinoma cells by decreasing PCNA levels, and induced apoptosis by up-regulating the expression of Bax and down-regulating the expression of Bcl-2. Previous studies have shown statistically significant differences in PCNA expression between normal gastric mucosa and gastric carcinoma. During the transition from normal gastric mucosa to gastric carcinoma, PCNA expression gradually increases 20. In the TCGA database, the expression of PCNA in gastric carcinoma tissues is higher than that in normal tissues, further confirming that downregulation of PCNA levels can inhibit gastric carcinoma cells proliferation. In addition, Anagnostopoulos GK et al. found that low expression of Bax in gastric carcinoma was associated with low 5-year survival and poor clinical prognosis 21.

In the 1965s, Laurén suggested that gastric carcinoma could be classified into intestinal and diffuse types according to the degree of differentiation and biological behavior 22. Intestinal type gastric carcinomas are caused by gastric mucosal atrophy and intestinal metaplasia caused by chronic helicobacter pylori infection, while diffuse type gastric carcinomas are considered to be arise from an abnormality of stem-like cells 23. Recent studies show that the cancer stem cell (CSC) markers, such as ABCB1 and CD133, were expressed higher in the diffuse type than in the intestinal type of human gastric carcinoma. CSC markers were also expressed higher in the undifferentiated cell line HGC-27 than the moderately-poorly differentiated cell line SGC-7901, which indicated that HGC-27 exhibited typical characteristics of diffuse gastric carcinoma. Compared to intestinal type gastric adenocarcinomas, the poorly-differentiated diffuse type gastric carcinoma showed more advanced stages, such as lymph node metastasis and vascular invasion 24. Herein, our data showed that the proliferation of SGC-7901 and HGC-27 cells was inhibited by high concentration of TSLL, and undifferentiated HGC-27 cells were more sensitive to TSLL treatment. In addition, the apoptotic induction rate of HGC-27 was higher than SGC-7901 cells. These results demonstrated that TSLL has a strong anti-cancer effect on SGC-7901 and HGC-27 cells, and has potential therapeutic effect on malignant diffuse.

Chemotherapy resistance and high recurrence rate of diffuse type gastric carcinoma are largely dependent on the aggressive cancer behavior, which is also important for the treatment of poorly differentiated malignant cancers 25. There are two key factors during the process of cancer invasion and metastasis: MMP-2 and TIMP-1. It has been reported that high expression of epithelial MMP-2 in gastric carcinoma is associated with low survival, while aggressive gastric carcinoma is associated with low expression of TIMP 26, 27. In gastric carcinoma, either inhibition of MMP-2 or up-regulation of TIMP-1 expression has been reported to suppress of metastasis 28, 29. Initially, metastatic cancer cells need to detach from the primary tumor, migrate and invade the basement membrane. MMPs can facilitate stromal and vascular invasion of cancer cells through the degradation of the extracellular matrix, play a crucial role in cancer metastasis steps 30. Here, TSLL successfully suppressed the migration and invasion of gastric carcinoma cells by decreasing the expression of MMP-2 and elevating the expression of TIMP-1. Besides, it has reported that MMP-2 and TIMP-1 are important regulatory proteins in EMT 31, 32. The critical mechanism of cancer progression and metastasis is the re-activation of the EMT 33, 34. However, another interesting observation showed that TSLL had no significant effect on EMT in gastric carcinoma cells which indicate TSLL might inhibit the invasion and metastasis of cancer by inhibiting other mechanisms besides EMT. Combined with the high sensitivity of undifferentiated gastric carcinoma cells to the anti-tumor effect of TSLL, the inhibitory effect of TSLL on invasion and migration of gastric carcinoma might be mediated by targeting cancer stem cells and inhibiting the enrichment of cancer stem cells 35, 36.

In conclusion, TSLL exerts its anticancer activity by inhibiting cell proliferation, migration and invasion, and inducing apoptosis in gastric carcinoma cells. Moreover, TSLL exhibit a stronger anticancer activity in undifferentiated gastric carcinoma cells, contributed to the treatment of diffuse-type gastric carcinoma. Future studies will be needed to on the potential mechanism of interaction between gastric carcinoma cell and TSLL, and the anti-gastric carcinoma effect of TSLL needs to be verified through in vivo animal experiments. Thus, TSLL may be used as an alternative or adjuvant to chemotherapeutic agents in gastric carcinoma treatment to reduce drug resistance and prognostic risk, and provide an attractive anticancer strategy for targeting diffuse-type gastric carcinoma.

## References

[1] Bray F, Ferlay J, Soerjomataram I (2018). Global cancer statistics 2018: GLOBOCAN estimates of incidence and mortality worldwide for 36 cancers in 185 countries. CA Cancer J Clin.

[2] Yoo C, Ryu MH, Park YS (2015). Intraoperatively assessed macroscopic serosal changes in patients with curatively resected advanced gastric cancer: clinical implications for prognosis and peritoneal recurrence. Ann Surg Oncol.

[3] Zong L, Abe M, Seto Y (2016). The challenge of screening for early gastric cancer in China. Lancet.

[4] Munafo JP (2015). Chemistry and biological activity of steroidal glycosides from the Lilium genus. Nat Prod Rep.

[5] Zhou ZL, Feng ZC, Fu CY (2012). Steroidal and phenolic glycosides from the bulbs of Lilium pumilum DC and their potential Na+/K+ ATPase inhibitory activity. Molecules.

[6] Wang T, Huang H, Zhang Y (2015). Role of effective composition on antioxidant, anti-inflammatory, sedative-hypnotic capacities of 6 common edible Lilium varieties. J Food Sci.

[7] Li W, Wang Z, Zha L (2017). HMGA2 regulates epithelial-mesenchymal transition and the acquisition of tumor stem cell properties through TWIST1 in gastric cancer. Oncol Rep.

[8] Karimian A, Ahmadi Y (2016). Multiple functions of p21 in cell cycle, apoptosis and transcriptional regulation after DNA damage. DNA Repair (Amst).

[9] Parveen A, Akash MS, Rehman K (2016). Dual Role of p21 in the Progression of Cancer and Its Treatment. Crit Rev Eukaryot Gene Expr.

[10] Hassan M, Watari H, AbuAlmaaty A (2014). Apoptosis and molecular targeting therapy in cancer. Biomed Res Int.

[11] Yao Z, Yuan T, Wang H (2017). MMP-2 together with MMP-9 overexpression correlated with lymph node metastasis and poor prognosis in early gastric carcinoma. Tumour Biol.

[12] Zheng H, Takahashi H, Murai Y (2006). Expressions of MMP-2, MMP-9 and VEGF are closely linked to growth, invasion, metastasis and angiogenesis of gastric carcinoma. Anticancer Res.

[13] Alpízar-Alpízar W, Laerum OD, Christensen IJ (2016). Tissue Inhibitor of Metalloproteinase-1 Is Confined to Tumor-Associated Myofibroblasts and Is Increased With Progression in Gastric Adenocarcinoma. J Histochem Cytochem.

[14] Wang S, Han H, Hu Y (2018). MicroRNA-130a-3p suppresses cell migration and invasion by inhibition of TBL1XR1-mediated EMT in human gastric carcinoma. Mol Carcinog.

[15] Yoon C, Cho SJ, Chang KK (2017). Role of Rac1 Pathway in Epithelial-to-Mesenchymal Transition and Cancer Stem-like Cell Phenotypes in Gastric Adenocarcinoma. Mol Cancer Res.

[16] Gonzalez DM (2014). Signaling mechanisms of the epithelial-mesenchymal transition. Sci Signal.

[17] Kanda K (2017). [The Necessity and the Current Status of Safe Handling of Anticancer Drugs]. Gan To Kagaku Ryoho.

[18] D'Alterio C, Scala S, Sozzi G (2019). Paradoxical effects of chemotherapy on tumor relapse and metastasis promotion. Semin Cancer Biol.

[19] Kinghorn AD, DE Blanco EJ, Lucas DM (2016). Discovery of Anticancer Agents of Diverse Natural Origin. Anticancer Res.

[20] Hu L, Li HL, Li WF (2017). Clinical significance of expression of proliferating cell nuclear antigen and E-cadherin in gastric carcinoma. World J Gastroenterol.

[21] Anagnostopoulos GK, Stefanou D, Arkoumani E (2007). Expression of Bax protein in gastric carcinomas. A clinicopathological and immunohistochemical study. Acta Gastroenterol Belg.

[22] LAUREN P, THE TWO HISTOLOGICAL MAIN TYPES OF GASTRIC CARCINOMA (1965). DIFFUSE AND SO-CALLED INTESTINAL-TYPE CARCINOMA. AN ATTEMPT AT A HISTO-CLINICAL CLASSIFICATION. Acta Pathol Microbiol Scand.

[23] Ming SC (1998). Cellular and molecular pathology of gastric carcinoma and precursor lesions: A critical review. Gastric Cancer.

[24] Marqués-Lespier JM, González-Pons M (2016). Current Perspectives on Gastric Cancer. Gastroenterol Clin North Am.

[25] Nakamura R, Omori T, Mayanagi S (2019). Risk of lymph node metastasis in undifferentiated-type mucosal gastric carcinoma. World J Surg Oncol.

[26] Nakata B, Muguruma K, Hirakawa K (1998). Predictive value of Bcl-2 and Bax protein expression for chemotherapeutic effect in gastric cancer. A pilot study. Oncology.

[27] Zhang M, Zhu GY, Gao HY (2011). Expression of tissue levels of matrix metalloproteinases and tissue inhibitors of metalloproteinases in gastric adenocarcinoma. J Surg Oncol.

[28] Gurgel DC, Valença-Junior JT, Dornelas CA (2015). Immunoexpression of metalloproteinases 2 and 14 and TIMP-2 inhibitor in main types of primary gastric carcinomas and lymph node metastasis. Pathol Oncol Res.

[29] Peduk S, Dincer M, Tatar C (2018). THE ROLE OF SERUM CK-18, MMP-9 AND TIPM-1 LEVELS IN PREDICTING R0 RESECTION IN PATIENTS WITH GASTRIC CANCER. Arq Bras Cir Dig.

[30] Löffek S, Schilling O (2011). Series "matrix metalloproteinases in lung health and disease": Biological role of matrix metalloproteinases: a critical balance. Eur Respir J.

[31] Duong TD (2004). MMP-2 plays an essential role in producing epithelial-mesenchymal transformations in the avian embryo. Dev Dyn.

[32] D'Angelo RC, Liu XW, Najy AJ (2014). TIMP-1 via TWIST1 induces EMT phenotypes in human breast epithelial cells. Mol Cancer Res.

[33] Chaffer CL, San Juan BP, Lim E (2016). EMT, cell plasticity and metastasis. Cancer Metastasis Rev.

[34] Singh M, Yelle N, Venugopal C.EMT (2018). Mechanisms and therapeutic implications. Pharmacol Ther.

[35] Nguyen DX, Bos PD (2009). Metastasis: from dissemination to organ-specific colonization. Nat Rev Cancer.

[36] Peitzsch C, Tyutyunnykova A, Pantel K (2017). Cancer stem cells: The root of tumor recurrence and metastases. Semin Cancer Biol.

